# Editorial: Intracellular transition metal homeostasis in plants and algae

**DOI:** 10.3389/fpls.2024.1430708

**Published:** 2024-05-22

**Authors:** Ádám Solti, Brigitta Tóth, Marie-Theres Hauser

**Affiliations:** ^1^ Department of Plant Physiology and Molecular Plant Biology, Eötvös Loránd University, Budapest, Hungary; ^2^ Institute of Food Science, University of Debrecen, Debrecen, Hungary; ^3^ Department of Applied Genetics and Cell Biology, University of Natural Resources and Life Sciences, Vienna, Austria

**Keywords:** epigenetics, heavy metal interactions, metalloproteins, metal uptake of organelles, transition metal sensing, transition metal signaling

## 1 Editorial overview

Plants established a highly structured regulatory network managing homeostasis of the essential transition metal which are widely employed as cofactors of redox enzymes. In the past century, dedicated transport systems have been identified for the uptake and translocation of essential transition metals (Eide et al., 1996; Curie et al., 2000). The current challenge in our understanding is how regulatory and intracellular transport processes contribute to the balanced metal homeostasis of plants.

Iron is the most abundant transition metal in all organisms and thus the most widely studied one in plants (Jeong, 2023). Vélez-Bermúdez and Schmidt review the knowledge of the past decade on Fe sensing and regulation. Although Fe sensing of mammalian cells is long known, the analogous mechanism of plant cells has a long debate. In plants, hemerythrin domain-containing proteins were found to be the key Fe sensors: both monocot and dicot Fe sensors (HRZ; BTS, respectively) express E3 ubiquitin ligase activity by their RING domain. This domain indeed shares a functional analogy to the mammalian Fe sensor FBXL5. The authors also question whether multiple Fe sensor systems in plant cells could orchestrate responses. According to current information, this question cannot be answered without any doubts. Downstream elements of the currently accepted HRZ/BTS systems are transcription factors of the bHLH family. Spielmann et al. review this complex cascade of bHLH transcription factors including their target genes involved in Fe uptake, mobilization and storage. Spielman et al. also present the current knowledge of alternative splicing upon Fe deficiency and the effect of non-Fe-metals on the post-translational control of the two well-characterized Fe transporters, NRAMP1 and IRT1. Lacking knowledge relates to the effect of post-translational modifications on the activity of transporters, their removal from the plasma membrane and their cellular polarity for directional uptake.

Loading of Fe into plastids is highly important in photosynthetically active cells. Since the discovery of the plastid localized FRO7 (Jeong et al., 2008) a light-dependent, reduction-based plastidial Fe uptake (Solti et al., 2012) was considered. Fe uptake in etioplasts is essential for the transformation towards chloroplasts and accumulation of the photosynthetic electron transport chain members. Yet, Fe acquisition in the absence of light cannot be a light-dependent process. According to *in vivo* Fe uptake and ferric chelate reductase activity of chloroplast envelope membranes presented by Sági-Kazár et al., the Fe uptake in etioplasts operates as a reduction-based process.

Zn homeostasis is also tightly modulated to avoid oxidative stress and cytotoxic interactions. Moreover, Zn is required for multiple chloroplast functions (Hübner and Haase, 2021). To achieve Zn homeostasis, a similar diversity of Zn managing proteins is expected as for Fe management. Zhang et al. investigated two *Arabidopsis* nucleotide-dependent metallochaperones ZNG1 & 2, orthologues to human and fungal ZNG1s. Based on structural modelling, protein localization, RNA-seq, and proteomic data, they found that ZNG1 interacts with the methionine aminopeptidases, MAP1A, and serves as Zn transferase for AtMAP1A. The target(s) of ZNG2s were presumed to be conserved plastid-localized proteins but need to be identified.

Balancing intracellular transition metal homeostasis involves dynamic genomic and transcriptional regulation. Evidences are increasing that among others altered Fe homeostasis changes the epigenetic regulation of Fe responses (Shafiq et al., 2020; Sun et al., 2021). Along with DNA methylation, the modification of histones represents a second platform of epigenetic regulations. Under Fe excess, repressive lysine methylation occurs on H3 histones leading to the suppression of Fe uptake (Séré and Martin, 2020). Non-essential metals and metalloids are serious threats to intracellular essential transition metal homeostasis by impacting, among others, protein folding, redox balance, and the incorporation of cofactors into metalloproteins. Chmielowska-Bąk et al. review the findings of non-essential metals and metalloids on the epigenetic regulation. The importance of their summary is that comprehensive reviews of the plant’s epigenetic responses to metals are still scarce. Although the information on epigenetic and epitranscriptomic alterations upon non-essential metal stresses is controversial, the higher methylation status and the activation of DNA methyltransferases, could result in a higher resilience of the nuclear DNA against damages, and could also affect the expression pattern of transporters, changes which would correlate with a higher tolerance against non-essential metals and metalloids.

## 2 Conclusions and future perspectives

Metal homeostasis is essential for development and environmental adaptation. In the intracellular transition metal homeostasis, characterization of Fe sensors, transporters, and transcription factor interactions are among the most important achievements. Indeed, sensing mechanisms for non-Fe metals and proposed additional Fe sensing systems are not yet completely resolved. Knowledge is slowly rising on the regulatory mechanisms at the epigenome and epitranscriptome level controlling the stability of the intracellular transition metal homeostasis. Future understanding of these mechanisms will reveal how stress memory, environmental adaptation, and intracellular interactions are induced by transition metals. Over molecular approaches, more data are needed from physical techniques that allow visualization of transition metals at tissue, cell, and intracellular levels. These include X-ray absorption and emission spectroscopy for the analysis of biological metal complexes (among others μXANES, EXAFS) and the distribution of transition metals (among others μXRF, EDX, PIXE), respectively. For the detection of transition metals in metalloprotein improved methods are needed such as among others HPLC-ICP-MS based techniques. Subcellular, and quantitative proteomic approaches are also of a growing importance. Future advancements in proteomic methodologies will facilitate the comprehensive characterization of the plant metalloproteome. Integrating multi-omics datasets will deeper the insight into the mechanisms governing plant metal homeostasis and enables to develop strategies for enhancing metal nutrition, stress tolerance, and crop productivity.

## Author contributions

ÁS: Writing – review & editing, Writing – original draft, Funding acquisition, Conceptualization. BT: Writing – review & editing, Writing – original draft, Conceptualization. M-TH: Writing – review & editing, Writing – original draft, Funding acquisition, Conceptualization.
